# Combining Biomarkers to Improve Diagnostic Accuracy in Detecting Diseases With Group‐Tested Data

**DOI:** 10.1002/sim.10230

**Published:** 2024-10-07

**Authors:** Jin Yang, Wei Zhang, Paul S. Albert, Aiyi Liu, Zhen Chen

**Affiliations:** ^1^ Biostatistics and Bioinformatics Branch *Eunice Kennedy Shriver* National Institute of Child Health and Human Development, National Institutes of Health Bethesda Maryland USA; ^2^ Academy of Mathematics and Systems Science Chinese Academy of Sciences Beijing China; ^3^ Biostatistics Branch, Division of Cancer Epidemiology and Genetics National Cancer Institute, National Institutes of Health Bethesda Maryland USA

**Keywords:** AUC, differential misclassification, joint model, multiple biomarkers

## Abstract

We consider the problem of combining multiple biomarkers to improve the diagnostic accuracy of detecting a disease when only group‐tested data on the disease status are available. There are several challenges in addressing this problem, including unavailable individual disease statuses, differential misclassification depending on group size and number of diseased individuals in the group, and extensive computation due to a large number of possible combinations of multiple biomarkers. To tackle these issues, we propose a pairwise model fitting approach to estimating the distribution of the optimal linear combination of biomarkers and its diagnostic accuracy under the assumption of a multivariate normal distribution. The approach is evaluated in simulation studies and applied to data on chlamydia detection and COVID‐19 diagnosis.

## 1 Introduction

In biomedical and epidemiological studies, diagnostic biomarkers are often used to distinguish diseased individuals from healthy ones in a potentially fast and economical way. For biomarkers with continuous results, the receiver operating characteristic (ROC) curve is a standard statistical tool to evaluate and compare their accuracy, consider Hanley and McNeil [1], Wieand et al. [2], Zou and Hall [3], Yang, Lu, and Zhao [4], and Yin and Tian [5], among others. The ROC curve of a biomarker is a plot of its sensitivity versus 1 minus specificity. The area or partial area under the ROC curve (AUC or pAUC) is a popular summary index, with large values indicating high diagnostic accuracy.

Although widely used, a single biomarker is usually not accurate enough and may incur considerable classification errors. It can be beneficial to use multiple biomarkers simultaneously so that one may obtain a combined biomarker with improved diagnostic accuracy. Under the assumption of multivariate normality, Su and Liu [6] derived the optimal linear combination of multiple biomarkers based on AUC. Following their work, Liu, Schisterman, Zhu [7] developed an optimal linear combination method by maximizing pAUC.

In many cases, it is possible that disease data are not available at individual subject level, due to cost and/or privacy consideration. In these situations, group testing has been advocated as an efficient alternative to reduce cost and protect confidentiality. The method was first proposed by Dorfman [8] to screen for syphilitic antigen in the U.S. army recruits based on pooled blood samples. Moreover, group testing has been shown to potentially improve statistical efficiency. Some works focus on the estimating the prevalence of disease, see Soberl and Elashoff [9], Le [10], Gastwirth and Hammick [11], Farrington [12], Hepworth [13], Hughes‐Oliver and Rosenberger [14], Turner et al. [15], Warasi et al. [16], Malinovsky, Haber, and Albert [17]; some focus on individual classification, see Chen and Swallow [18], Brookmeyer [19], Zhang et al. [20], Black et al. [21], Hepworth and Watson [22], Zhang et al. [23]; and others focus on multiplex assays, see Tebbs, McMahan, and Bilder [24], Li, Liu, Xiong [25], Warasi et al. [26], among others.

A practical scenario for which our method is particularly useful is when the conventional “gold standard” tests are prohibitively expensive and time‐consuming, making it desirable to develop less expensive and more convenient procedures, with reasonable accuracy. To the best of our knowledge, little work has been done in the literature on diagnostic biomarkers in the context of group testing scheme, Zhang et al. [27] proposed a nonparametric estimation approach for a single biomarker when only group‐based test results on the disease status are available. In the present paper, we proposed a cost‐effective alternative by combining multiple biomarkers that are less expensive, more convenient to measure, and offer higher diagnostic accuracy of the disease in the context of group testing. This problem has several challenges, including (1) unavailable individual disease statuses, (2) differential misclassification which may depend on the group size and the number of diseased individuals in the group [28], and (3) extensive computational demands due to the large number of possible combinations of multiple biomarkers. To tackle these issues, we propose to estimate the distributions of biomarkers in the diseased and non‐diseased populations based on the pairwise bivariate fitting procedure of Fieuws and Verbeke [29] and Kassahun–Yimer et al. [30]. Furthermore, we will construct an estimator of the ROC curve for the optimal linear combination of multiple biomarkers, providing a robust approach for quick and accurate disease diagnosis.

The article is organized as follows. We present the estimation strategies for the distributions of multiple biomarkers in Section 2 and the ROC curve of their optimal linear combination in Section 3. Extensive simulation studies are conducted in Section 4 to compare the statistical efficiency of estimators based on group and individual testings. In Section 5, we illustrate our methods with data on chlamydia detection from NHANES and COVID‐19 diagnosis from UK Biobank. Conclusions and further research directions are given in Section 6.

## 2 Methodology

### 2.1 Likelihood Function of the Multivariate Model

We assume the values of biomarkers can be observed on each individual subject. However, the binary disease status (e.g., disease and non‐disease) is only observed at group level, that is, the binary testing is only performed on pooled samples rather than individual sample. Our aim is to find the linear combination of the individual biomarkers that maximize the AUC.

To be specific, let$𝒳={\left(X_{1},\ldots ,X_{m}\right)}^{T}$ denote the measurements ofm biomarkers with the distributionH and the probability density function (pdf)h, and the symbol “T” standing for the transpose of a vector or matrix. LetD be the true binary status of a disease with the prevalencePr(D=1)=p andK be the observed disease status with specificity$\delta_{0}=\mathrm{Pr}(K=0|D=0)$ and sensitivity$\delta_{1}=\mathrm{Pr}(K=1|D=1)$, respectively. In this article, our main interest is to estimate the diagnostic accuracy of multiple biomarkers. To this end, we need to estimate the conditional distributions of𝒳 in non‐diseased and diseased population, which are denoted byF andG, respectively. Letf andg be the corresponding density functions ofF andG. It is easy to getH=(1−p)F+pG.

Suppose thatF andG are multivariate normal distributions, that is,

(1) $$\begin{array}{ll} & {𝒳}^{T}|D=0∼N\left(\mu^{\left(0\right)},\sum^{\left(0\right)}\right)=F\\ & {𝒳}^{T}|D=1∼N\left(\mu^{\left(1\right)},\sum^{\left(1\right)}\right)=G \end{array}$$

with mean vectors$\mu^{\left(0\right)}={\left(\mu_{1}^{\left(0\right)},\ldots ,\mu_{m}^{\left(0\right)}\right)}^{T}$ and$\mu^{\left(1\right)}={\left(\mu_{1}^{\left(1\right)},\ldots ,\mu_{m}^{\left(1\right)}\right)}^{T}$, and covariance matrices

$$\sum^{\left(0\right)}=\left\{\sigma_{tt^{\prime }}^{\left(0\right)}\right\},\sum^{\left(1\right)}=\left\{\begin{array}{c} \sigma_{tt^{\prime }}^{\left(1\right)} \end{array}\right\},\;\text{where}\;t,t^{\prime }=1,\ldots ,m$$

ConsiderN subjects that are randomly divided inton groups with sizes$J_{i},i=1,\ldots ,n$, where$J_{1}+\cdots +J_{n}=N$. The vector𝒳 can be observed on each subject, yielding the observation$\left\{X_{ij,1},\ldots ,X_{ij,m},j=1,\ldots ,J_{i},i=1,\ldots ,n\right\}$. Denote${𝕏}_{i,t}=(X_{i1,t},\ldots ,X_{iJ_{i},t}),t=1,\ldots ,m$. Thus$\left({𝕏}_{i,1},\ldots ,{𝕏}_{i,m})=({\left(X_{i1,1},\ldots ,X_{iJ_{i},1}\right)}^{T},\ldots ,{\left(X_{i1,m},\ldots ,X_{iJ_{i},m}\right)}^{T}\right)$, fori=1,…,n. Denote the group‐tested results of the disease by${\tilde{K}}_{i},i=1,\ldots ,n$, and let$D_{ij}$ be the true disease status of thejth subject in theith group and define$\tilde{D_{i}}=\max\{D_{i1},\ldots ,D_{iJ_{i}}\}$ which is the true disease status of groupi. For each groupi, we assume that the specificity of the test remains unchanged, that is,$\mathrm{Pr}(\tilde{K_{i}}=0|\tilde{D_{i}}=0)=\delta_{0}$, and the sensitivity is differential which depends on the group size$J_{i}$ and the number of diseased subjects$d_{i}$ in the group, see Haber, Malinovsky, and Albert [28], Hwang [31], and Hung and Swallow [32], denoted by$\mathrm{Pr}(\tilde{K_{i}}=1|\tilde{D_{i}}=1)=\overset{*}{\underset{1}{\delta }}(J_{i},d_{i})$.

Given the true group disease status${\tilde{D}}_{i}$, we assume the biomarkers' level$\left({𝕏}_{i,1},\ldots ,{𝕏}_{i,m}\right)$ and group‐tested results${\tilde{K}}_{i}$ are independent. Thus, we can obtain

$$\begin{array}{l} h\{({𝕏}_{i,1},\ldots ,{𝕏}_{i,m})|\tilde{K}=k,\tilde{D}=d\}=h\{({𝕏}_{i,1},\ldots ,{𝕏}_{i,m})|\tilde{D}=d\} \end{array}$$

wherek,d∈{0,1}. Denote$h\{({𝕏}_{i,1},\ldots ,{𝕏}_{i,m}),{\tilde{K}}_{i}\}$ is the joint density function of$\left({𝕏}_{i,1},\ldots ,{𝕏}_{i,m}\right)$ and${\tilde{K}}_{i}$. LetI(·) be an indicator function. Then the likelihood function for the observed data is given by

(2) $$\begin{array}{ll} L & =\overset{n}{\underset{i=1}{\prod }}h{\left\{({𝕏}_{i,1},\ldots ,{𝕏}_{i,m}),{\tilde{K}}_{i}=0\right\}}^{I({\tilde{K}}_{i}=0)}\\ & \;\overset{n}{\underset{i=1}{\prod }}h{\left\{({𝕏}_{i,1},\ldots ,{𝕏}_{i,m}),{\tilde{K}}_{i}=1\right\}}^{I({\tilde{K}}_{i}=1)} \end{array}$$

where

(3) $$\begin{array}{ll} & h\left\{({𝕏}_{i,1},\ldots ,{𝕏}_{i,m}),{\tilde{K}}_{i}=0\right\}\\ & \;=\delta_{0}{\left(1-p\right)}^{J_{i}}\overset{J_{i}}{\underset{j=1}{\prod }}f(X_{ij,1},\ldots ,X_{ij,m})\\ & \;+\underset{\{\nu_{1},\ldots ,\nu_{J_{i}}\}\in 𝒜}{\sum }\left\{\overset{J_{i}}{\underset{j=1}{\prod }}f{\left(X_{ij,1},\ldots ,X_{ij,m}\right)}^{1-\nu_{j}}g{\left(X_{ij,1},\ldots ,X_{ij,m}\right)}^{\nu_{j}}\right\}\\ & \;\times \left\{1-\delta_{1}^{*}(J_{i},d_{i})\right\}p^{d_{i}}{\left(1-p\right)}^{J_{i}-d_{i}}\\ & h\left\{({𝕏}_{i,1},\ldots ,{𝕏}_{i,m}),{\tilde{K}}_{i}=1\right\}\\ & \;=(1-\delta_{0}){\left(1-p\right)}^{J_{i}}\overset{J_{i}}{\underset{j=1}{\prod }}f(X_{ij,1},\ldots ,X_{ij,m})\\ & \;+\underset{\{\nu_{1},\ldots ,\nu_{J_{i}}\}\in 𝒜}{\sum }\left\{\overset{J_{i}}{\underset{j=1}{\prod }}f{\left(X_{ij,1},\ldots ,X_{ij,m}\right)}^{1-\nu_{j}}g{\left(X_{ij,1},\ldots ,X_{ij,m}\right)}^{\nu_{j}}\right\}\\ & \;\delta_{1}^{*}(J_{i},d_{i})p^{d_{i}}{\left(1-p\right)}^{J_{i}-d_{i}} \end{array}$$

where$𝒜=\{\{\nu_{1},\ldots ,\nu_{J_{i}}\}\;|\;\nu_{j}\in \{0,1\},\;\nu_{1}+\cdots +\nu_{J_{i}}=d_{i}>0\}$.

In the present article, we focus on equal group size, which is most commonly used choice in practice. The results can be extended unequal size. Assume$J_{1}=\cdots =J_{n}=N/n≜J$. With the likelihood functionL, we can use the maximum likelihood estimation (MLE) approach to estimateF andG. The log‐likelihood function for$\left\{{\left(({𝕏}_{i,1}^{T},\ldots ,{𝕏}_{i,m}^{T}),{\tilde{K}}_{i}\right)}^{T}:i=1,\ldots ,n\right\}$ is given by

(4) $$\begin{array}{ll} & l_{n}\left\{p;\mu^{\left(0\right)},\sum^{\left(0\right)},\mu^{\left(1\right)},\sum^{\left(1\right)}\right\}\\ & \;=\overset{n}{\underset{i=1}{\sum }}I({\tilde{K}}_{i}=1)\log\left[\left(1-\delta_{0}){\left(1-p\right)}^{J}\overset{J}{\underset{j=1}{\prod }}f(X_{ij,1},\ldots ,X_{ij,m}\right)\right.\\ & \;+\underset{\{\nu_{1},\ldots ,\nu_{J_{i}}\}\in 𝒜}{\sum }\left\{\overset{J}{\underset{j=1}{\prod }}f{\left(X_{ij,1},\ldots ,X_{ij,m}\right)}^{1-\nu_{j}}g{\left(X_{ij,1},\ldots ,X_{ij,m}\right)}^{\nu_{j}}\right\}\\ & \;\left.\delta_{1}^{*}(J,d)p^{d}{\left(1-p\right)}^{J-d}\right]\\ & \;+\overset{n}{\underset{i=1}{\sum }}I({\tilde{K}}_{i}=0)\log\left[\delta_{0}{\left(1-p\right)}^{J}\overset{J}{\underset{j=1}{\prod }}f(X_{ij,1},\ldots ,X_{ij,m})\right.\\ & \;+\underset{\{\nu_{1},\ldots ,\nu_{J_{i}}\}\in 𝒜}{\sum }\left\{\overset{J}{\underset{j=1}{\prod }}f{\left(X_{ij,1},\ldots ,X_{ij,m}\right)}^{1-\nu_{j}}g{\left(X_{ij,1},\ldots ,X_{ij,m}\right)}^{\nu_{j}}\right\}\\ & \;\left.\left\{1-\delta_{1}^{*}(J,d)\right\}p^{d}{\left(1-p\right)}^{J-d}\right] \end{array}$$

where

$$\begin{array}{ll} & f(X_{ij,1},\ldots ,X_{ij,m})\\ & \;=\frac{1}{{\left(2\pi \right)}^{m/2}{\left\{\det(\sum^{\left(0\right)})\right\}}^{1/2}}\\ & \;\exp\left[-\frac{1}{2}{\left\{{𝒳}^{\left(0\right)}-\mu^{\left(0\right)}\right\}}^{T}{\left\{\sum^{\left(0\right)}\right\}}^{-1}\left\{{𝒳}^{\left(0\right)}-\mu^{\left(0\right)}\right\}\right]\\ & g(X_{ij,1},\ldots ,X_{ij,m})\\ & \;=\frac{1}{{\left(2\pi \right)}^{m/2}{\left\{\det(\sum^{\left(1\right)})\right\}}^{1/2}}\\ & \;\exp\left[-\frac{1}{2}{\left\{{𝒳}^{\left(1\right)}-\mu^{\left(1\right)}\right\}}^{T}{\left\{\sum^{\left(1\right)}\right\}}^{-1}\left\{{𝒳}^{\left(1\right)}-\mu^{\left(1\right)}\right\}\right] \end{array}$$

### 2.2 Estimation Procedure

It is not easy to estimate the parameters by directly maximizing (4), especially whenm is large. To address this issue, we employ the pairwise fitting approach which uses the pairwise models to represent the original multivariate model, see Fieuws and Verbeke [29], Kassahun‐Yimer et al. [30], and Fieuws, Verbeke, and Molenberghs [33]. The general idea is that the parameters in the full multivariate model can be identified from all pairwise models (the bivariate model for each pair of biomarkers). Related to pseudo‐likelihood estimation (Besag [34]), the estimation procedure can be summarized as the following:

- Instead of maximizing the log‐likelihood of the multivariate model, log‐likelihoods of the following form will be maximized separately
(5) $$\begin{array}{l} \overset{n}{\underset{i=1}{\sum }}l_{rs,i}(\Omega_{r,s}) \end{array}$$
  wherer=1,…,m−1,s=r+1,…,m, and$\Omega_{r,s}$ is the vector containing all parameters in the bivariate model corresponding to the specific pair(r,s), and the log‐likelihood function for therth andsth biomarkers can be written as
(6) $$\begin{array}{l} l_{rs}\left\{p;\mu_{r}^{\left(0\right)},\mu_{s}^{\left(0\right)},\mu_{r}^{\left(1\right)},\mu_{s}^{\left(1\right)},\sum_{r}^{\left(0\right)},\sum_{s}^{\left(0\right)},\sum_{r}^{\left(1\right)},\sum_{s}^{\left(1\right)}\right\} \end{array}$$
  which is the same as (4) with$f(X_{ij,1},\ldots ,X_{ij,m})$ and$g(X_{ij,1},\ldots ,X_{ij,m})$ replaced by$f(X_{ij,r},X_{ij,s})$ and$g(X_{ij,r},X_{ij,s})$, respectively.
- For simplicity, (5) can be rewritten as$\sum_{i=1}^{n}l_{p,i}(\Omega_{p})$ withp=1,…,W. So we note that there are a total ofW=m(m−1)/2 possible pairs.
- Let${\hat{\Omega }}_{rs}$ be the maximizer of$l_{n}(\Omega_{rs})$ with respect to$\Omega_{rs}$ and$\hat{\Omega }$ be the stacked vector containing all pair‐specific parameter vectors${\hat{\Omega }}_{rs}$. Then, the final estimator for each parameter inΩ can be obtained by averaging all pair‐specific estimators in$\hat{\Omega }$. The estimators ofF andG can be subsequently derived and are denoted by$\hat{F}$ and$\hat{G}$.

The above procedure has the advantage when it is impossible or too time consuming to fit, as summarized by Fieuws and Verbeke [29]: “the pairwise approach is able to yield unbiased estimators with robust variances which indicates that it can capture the true sampling variability.” It is possible that the approach may result in an estimator of covariance matrix, that is not positive definite. That is because we are averaging pairwise covariance, particularly in large dimension. When the correlation are high, a non‐positive definite covariance can occur. In such a case, we can shrink the off diagonal element toward zero resulting in a positive definite covariance matrix, see Devlin, Gnanadesikan, and Kettenring [35].

## 3 ROC Curves and AUC

Without loss of generality, we assume that the values of the biomarkers in the diseased population tend to be larger than that in the non‐diseased population. Consider a biomarker that follows the normal distributions$N(\mu_{1}^{\left(1\right)},{\left\{\sigma_{1}^{\left(1\right)}\right\}}^{2})$ and$N(\mu_{1}^{\left(0\right)},{\left\{\sigma_{1}^{\left(0\right)}\right\}}^{2})$ in the diseased and non‐diseased population, respectively. The ROC curve of the biomarker is given by

(7) $$\begin{array}{l} \text{ROC}(q)=\Phi \{u_{1}-v_{1}\Phi^{-1}(1-q)\} \end{array}$$

for0≤q≤1, where$u_{1}=(\mu_{1}^{\left(1\right)}-\mu_{1}^{\left(0\right)})/\sigma_{1}^{\left(1\right)}$ and$v_{1}=\sigma_{1}^{\left(1\right)}/\sigma_{1}^{\left(0\right)}$,Φ is the cumulative distribution function of the standard normal distribution.

For the case of multiple biomarkers, Su and Liu [6] gave an explicit form for the best linear combinationβ, which has the form$c\times \mu^{T}{\left(\sum^{\left(1\right)}+\sum^{\left(0\right)}\right)}^{-1}$, wherec is the reciprocal of the first element of$\mu^{T}{\left(\sum^{\left(1\right)}+\sum^{\left(0\right)}\right)}^{-1}$ and$\mu =\mu^{\left(1\right)}-\mu^{\left(0\right)}$, see chapter 4 in Zou et al. [36]. Based on the results of Su and Liu [6], the ROC curve and AUC for the best linear combination of multiple biomarkers are given by

(8) $$\begin{array}{l} \text{ROC}(q)=\Phi \{u-v\Phi^{-1}(1-q)\} \end{array}$$

and

(9) $$\begin{array}{l} \text{AUC}=\Phi \left\{\sqrt{\mu^{T}{\left(\sum^{\left(0\right)}+\sum^{\left(1\right)}\right)}^{-1}\mu }\right\} \end{array}$$

where$u=\frac{\beta^{T}(\mu^{\left(1\right)}-\mu^{\left(0\right)})}{\sqrt{\beta^{T}\sum^{\left(0\right)}\beta }}$ and$v=\frac{\sqrt{\beta^{T}\sum^{\left(1\right)}\beta }}{\sqrt{\beta^{T}\sum^{\left(0\right)}\beta }}$.

## 4 Simulations

In evaluating the performance of our proposed approach through simulations, we have two goals in mind: (1) to compare multiple biomarkers versus single biomarker and (2) to compare group‐tested results versus individual‐tested results. Due to study budget and other constraints, it may not be always possible to include allN subjects in testing. As a result, we considered two scenarios when we approached the individual tested data, with the first using individual tested results of allN=nJ subjects (which are called *full individual testing*) and the second using a random sample of groupn from thenJ subjects (which are called *random individual testing*).

In this section, we consider three biomarkers. The group sizeJ is chosen from{1,2,5}, whereJ=1 corresponds to individual testing. The sensitivity$\delta_{1}$ and specificity$\delta_{0}$ were selected from{0.90,0.95,1.00}. We specified$\delta_{1}^{*}$ using the model of Hung and Swallow [32] as$\delta_{1}^{*}=\delta_{1}d/\{d+\lambda (J-d)\}$, whered represents the number of diseased individuals in a group, withλ=0.02. The prevalencep is set to be 0.01 or 0.02. For a given sample sizeN=nJ, we generated the random sample of the biomarkers$\left({𝕏}_{1}^{T},\ldots ,{𝕏}_{m}^{T}\right)$ and disease status$\tilde{K}$ using the following procedure. We first individually simulated the true disease status$\left\{D_{ij}:1\leq i\leq n;1\leq j\leq J\right\}$ for all subjects from a Bernoulli distribution with probabilityp. For the subjects withD=0, values of biomarkers were generated from the multivariate normal distribution with mean(0,0,0) and covariance matrix with diagonal elements 1, 1, and 1 and pairwise correlation coefficients 1/3, 1/3, and 1/3; otherwise, they were generated from the multivariate normal distribution with mean(1.6,1.5,1.2) and covariance matrix with diagonal elements$1.4^{2}$,$1.5^{2}$, and$1.3^{2}$ and pairwise correlation coefficients 0.5, 0.6, and 0.3, respectively. Then we randomly divided theN subjects inton groups of size J. We generated the group‐tested result ($\tilde{K}$) from a Bernoulli distribution with the probability$1-\delta_{0}$ for the groups with allD=0 and the probability$\delta_{1}^{*}$ for the groups with at least oneD=1. The true value of AUC for the three biomarkers and the best linear combination of the biomarkers are 0.8238, 0.7973, 0.7678, and 0.8579, respectively.

We assessed the proposed estimation approach under varying number of groups (*full individual* vs. *random individual testing*), group size (J=1,2,5), prevalence (p=0.01,0.02), and misclassification rate ($1-\delta_{0}=1-\delta_{1}=0,0.05,0.1$). For a more comprehensive comparison, we also consider the AUCs of individual biomarkers based on group‐tested data. The evaluations were based on commonly used criteria including bias, variance, 95% coverage probability (CP) and the average confidence interval length (ACIL). We used the bootstrap procedure which resamples the group‐level observations$\left\{({𝕏}_{i,1},\ldots ,{𝕏}_{i,m}),{\tilde{K}}_{i}):i=1,\ldots ,n\right\}$ with replacement to estimate CP and ACIL. Throughout, 200 simulation data were obtained and 300 bootstrap replicates were generated within each simulation.

In the scenario of *full individual testing*, we fixed the total number of subjects atN=15000. Table 1 presents the performance of the prevalence estimator in a finite sample setting. Regardless of the true prevalence level (across the two row blocks), number of biomarkers (across the columns), or misclassification rate (across the rows within each row block), the estimates are all close to the true values, with coverage probabilities close to the nominal level. The estimator exhibits increased statistical efficiency (indicated by smaller variance or shorter ACIL) as the misclassification error ($1-\delta_{0}$ and$1-\delta_{1}$) decreases, resulting in consistent findings with Zhang et al. [27] who focused on a single biomarker. For example, when prevalence isp=0.02, misclassification rate are both equal to 0.1 ($\delta_{1}=\delta_{0}=0.9$), and the group size isJ=5, the relative efficiency of the estimator under our proposed approach is about 0.77 (0.0317/0.0410) in comparison toJ=1. Moreover, the estimators based on the combined biomarker can improve the efficiency over that based on a single biomarker [27]. For example, whenp=0.02,$\delta_{1}=\delta_{0}=0.9$, andJ=5, the relative efficiency of the estimator based on the combined biomarker is 0.76, which is less than 1. In essence, utilizing multiple biomarkers with group‐tested data achieves double efficiency gains, one from group‐tested data in the presence of misclassification and the other from multiple biomarkers.

**TABLE 1:** Simulation results for the prevalence estimator based on the group and *full individual testing* approaches: estimate (Est), bias (Bias), variance (Var), coverage probability (CP) and average confidence interval length (ACIL) of the estimators for the individual (B‐1, B‐2, B‐3) and combined biomarkers.

		p=0.01															
		Individual												Combined			
		B‐1				B‐2				B‐3							
$\delta_{0}=\delta_{1}$	J	Est	Bias	Var	CP (ACIL)	Est	Bias	Var	CP (ACIL)	Est	Bias	Var	CP (ACIL)	Est	Bias	Var	CP (ACIL)
0.90	1	0.9979	−0.0021	0.0672	94.00% (0.0107)	0.9907	−0.0093	0.0674	92.00% (0.0108)	1.0002	0.0002	0.0880	91.50% (0.0118)	1.0074	0.0074	0.0258	95.00% (0.0069)
	2	0.9915	−0.0085	0.0470	95.50% (0.0090)	0.9835	−0.0165	0.0502	93.00% (0.0089)	0.9941	−0.0059	0.0449	95.50% (0.0093)	1.0066	0.0066	0.0245	95.50% (0.0065)
	5	1.0059	0.0059	0.0310	94.00% (0.0070)	1.0003	0.0003	0.0309	95.00% (0.0070)	1.0067	0.0067	0.0316	96.50% (0.0072)	1.0107	0.0107	0.0238	93.00% (0.0059)
0.95	1	1.0063	0.0063	0.0414	90.45% (0.0076)	0.9863	−0.0137	0.0422	91.96% (0.0078)	1.0008	0.0008	0.0423	94.47% (0.0084)	1.0041	0.0041	0.0201	95.98% (0.0058)
	2	1.0001	0.0001	0.0254	96.50% (0.0065)	0.9968	−0.0032	0.0267	95.00% (0.0065)	1.0000	0.0000	0.0252	95.50% (0.0066)	1.0086	0.0086	0.0180	94.00% (0.0053)
	5	1.0106	0.0106	0.0160	94.95% (0.0052)	1.0075	0.0075	0.0153	96.46% (0.0052)	1.0089	0.0089	0.0154	95.96% (0.0052)	1.0129	0.0129	0.0137	94.44% (0.0047)
1.00	1	1.0063	0.0063	0.0074	95.50% (0.0031)	1.0063	0.0063	0.0073	95.50% (0.0031)	1.0060	0.0060	0.0073	95.50% (0.0031)	1.0144	0.0144	0.0067	95.00% (0.0030)
	2	1.0065	0.0065	0.0075	96.00% (0.0032)	1.0064	0.0064	0.0075	95.50% (0.0032)	1.0064	0.0064	0.0075	95.50% (0.0032)	1.0137	0.0137	0.0070	95.50% (0.0031)
	5	1.0078	0.0078	0.0080	94.97% (0.0033)	1.0073	0.0073	0.0080	94.47% (0.0034)	1.0077	0.0077	0.0081	93.97% (0.0034)	1.0136	0.0136	0.0072	93.97% (0.0032)

		p=0.02															
		Individual												Combined			
		B‐1				B‐2				B‐3							
$\delta_{0}=\delta_{1}$	J	Est	Bias	Var	CP (ACIL)	Est	Bias	Var	CP (ACIL)	Est	Bias	Var	CP (ACIL)	Est	Bias	Var	CP (ACIL)
0.90	1	1.9949	−0.0051	0.0787	97.50% (0.0122)	1.9876	−0.0124	0.0826	94.50% (0.0121)	1.9965	−0.0035	0.0902	95.00% (0.0129)	1.9981	−0.0019	0.0410	95.00% (0.0086)
	2	2.0027	0.0027	0.0536	96.00% (0.0097)	1.9904	−0.0096	0.0572	95.00% (0.0099)	1.9992	−0.0008	0.0588	96.50% (0.0100)	2.0007	0.0007	0.0341	96.00% (0.0079)
	5	1.9995	−0.0005	0.0420	94.50% (0.0079)	1.9963	−0.0037	0.0419	93.50% (0.0079)	2.0045	0.0045	0.0419	94.00% (0.0079)	2.0077	0.0077	0.0317	94.00% (0.0070)
0.95	1	2.0024	0.0024	0.0559	91.50% (0.0087)	1.9897	−0.0103	0.0506	93.00% (0.0088)	2.0011	0.0011	0.0579	92.00% (0.0091)	1.9979	−0.0021	0.0341	94.50% (0.0069)
	2	1.9948	−0.0052	0.0335	93.50% (0.0073)	1.9918	−0.0082	0.0340	95.00% (0.0073)	1.9963	−0.0037	0.0339	93.50% (0.0073)	2.0021	0.0021	0.0277	92.50% (0.0064)
	5	1.9985	−0.0015	0.0236	96.00% (0.0062)	2.0010	0.0010	0.0243	95.50% (0.0062)	2.0014	0.0014	0.0246	95.50% (0.0063)	2.0066	0.0066	0.0220	94.50% (0.0058)
1.00	1	2.0005	0.0005	0.0139	94.50% (0.0044)	2.0002	0.0002	0.0139	94.00% (0.0044)	1.9998	−0.0002	0.0139	95.00% (0.0044)	2.0108	0.0108	0.0132	94.00% (0.0043)
	2	2.0006	0.0006	0.0141	96.00% (0.0045)	2.0008	0.0008	0.0142	95.50% (0.0045)	2.0011	0.0011	0.0142	95.00% (0.0045)	2.0097	0.0097	0.0133	95.00% (0.0044)
	5	1.9995	−0.0005	0.0165	92.50% (0.0047)	2.0002	0.0002	0.0163	93.50% (0.0047)	2.0007	0.0007	0.0166	93.00% (0.0047)	2.0084	0.0084	0.0155	93.00% (0.0046)

*Note*: Entries of Est and Bias are multiplied by 100, and entries of Var are multiplied by 10000 for better presentation.p is the prevalence,$\delta_{0}$ and$\delta_{1}$ are the specificity and sensitivity,J is the group size, and B‐1, B‐2, B‐3 stand for individual biomarker 1, 2, 3, respectively.

Table 2 summarizes the performance of the AUC estimator in a finite sample setting. As expected, combining biomarker (“Combined”) yields larger AUC estimates, indicating improved diagnostic accuracy compared to individual biomarkers (“Individual”). For example, whenp=0.01,$\delta_{0}=\delta_{1}=0.9$, andJ=2, the AUCs of the single biomarkers are 0.8304, 0.8096 and 0.7730, respectively, and the AUC of combined biomarker is 0.8721. Similar to the findings for the prevalence estimator, as the misclassification error decreases, the variance of the estimators decreases. Furthermore, the AUC estimator of the combined biomarkers is always more efficient than their single biomarker counterparts, as its variance is smaller than all those in the individual setting. Focusing on the estimators based on the combined biomarker, we can see that, when there is no misclassification ($\delta_{0}=\delta_{1}=1.00$), the superiority of group testing disappears, with yielding larger variance than that based on individual testing. Moreover, the AUC estimates based on group testing are close to that based on individual testing. However, when misclassification exists ($\delta_{0},\;\delta_{1}<1.00$), there is always an estimator based on group testing that yields smaller variance than the individual testing counterpart. For example, whenp=0.01,$\delta_{0}=\delta_{1}=0.95$, the AUC estimator achieves the smallest variance atJ=5. These results suggest that the AUC estimators based on group testing can be more efficient than those based on individual testing when the test is subject to misclassification.

**TABLE 2:** Simulation results for the AUC estimator based on the group and *full individual testing* approaches: estimate (Est), bias (Bias) and variance (Var), coverage probability (CP) and average confidence interval length (ACIL) of the estimators for the individual (B‐1, B‐2, B‐3) and combined biomarkers.

		p=0.01															
		Individual												Combined			
		B‐1				B‐2				B‐3							
$\delta_{0}=\delta_{1}$	J	Est	Bias	Var	CP (ACIL)	Est	Bias	Var	CP (ACIL)	Est	Bias	Var	CP (ACIL)	Est	Bias	Var	CP (ACIL)
0.90	1	0.8317	0.0079	0.0558	87.50% (0.2676)	0.8086	0.0113	0.0545	91.00% (0.2818)	0.7811	0.0133	0.0708	91.00% (0.3291)	0.8714	0.0135	0.0127	88.50% (0.1442)
	2	0.8304	0.0066	0.0426	91.00% (0.2546)	0.8096	0.0123	0.0615	86.00% (0.2557)	0.7730	0.0052	0.0666	84.00% (0.2860)	0.8721	0.0142	0.0144	86.50% (0.1403)
	5	0.8219	−0.0019	0.0406	89.50% (0.2309)	0.8087	0.0114	0.0418	88.50% (0.2347)	0.7742	0.0064	0.0580	89.00% (0.2834)	0.8721	0.0142	0.0116	91.00% (0.1392)
0.95	1	0.8302	0.0064	0.0371	86.43% (0.2135)	0.8057	0.0084	0.0413	89.95% (0.2314)	0.7792	0.0114	0.0455	90.45% (0.2540)	0.8702	0.0123	0.0108	89.45% (0.1318)
	2	0.8249	0.0011	0.0262	91.00% (0.2035)	0.8015	0.0042	0.0316	92.00% (0.2102)	0.7687	0.0009	0.0360	94.00% (0.2359)	0.8660	0.0081	0.0123	90.00% (0.1302)
	5	0.8231	−0.0007	0.0234	93.43% (0.1924)	0.7993	0.0020	0.0265	93.94% (0.1963)	0.7709	0.0031	0.0320	94.95% (0.2246)	0.8679	0.0100	0.0104	90.40% (0.1225)
1.00	1	0.8231	−0.0007	0.0042	91.00% (0.0760)	0.7963	−0.0010	0.0040	97.00% (0.0839)	0.7690	0.0012	0.0049	93.00% (0.0827)	0.8619	0.0040	0.0024	88.00% (0.0536)
	2	0.8264	0.0026	0.0057	94.00% (0.0898)	0.7969	−0.0004	0.0055	95.50% (0.0974)	0.7671	−0.0007	0.0076	94.00% (0.1043)	0.8622	0.0043	0.0028	91.50% (0.0620)
	5	0.8211	−0.0027	0.0093	96.98% (0.1234)	0.8029	0.0056	0.0098	91.96% (0.1276)	0.7682	0.0004	0.0154	91.96% (0.1491)	0.8621	0.0042	0.0047	89.45% (0.0784)

		p=0.02															
		Individual												Combined			
		B‐1				B‐2				B‐3							
$\delta_{0}=\delta_{1}$	J	Est	Bias	Var	CP (ACIL)	Est	Bias	Var	CP (ACIL)	Est	Bias	Var	CP (ACIL)	Est	Bias	Var	CP (ACIL)
0.90	1	0.8285	0.0047	0.0190	93.50% (0.1791)	0.8021	0.0048	0.0207	94.50% (0.1867)	0.7744	0.0066	0.0255	93.00% (0.2018)	0.8646	0.0067	0.0068	93.00% (0.1034)
	2	0.8212	−0.0026	0.0182	91.50% (0.1548)	0.8029	0.0056	0.0162	92.50% (0.1642)	0.7687	0.0009	0.0207	94.00% (0.1817)	0.8643	0.0064	0.0064	86.50% (0.0967)
	5	0.8187	−0.0051	0.0132	95.00% (0.1487)	0.8016	0.0043	0.0159	93.00% (0.1483)	0.7615	−0.0063	0.0213	91.50% (0.1809)	0.8635	0.0056	0.0048	93.00% (0.0904)
0.95	1	0.8248	0.0010	0.0128	92.00% (0.1381)	0.8002	0.0029	0.0123	95.00% (0.1420)	0.7729	0.0051	0.0134	96.00% (0.1508)	0.8628	0.0049	0.0051	90.00% (0.0858)
	2	0.8234	−0.0004	0.0101	94.00% (0.1253)	0.8011	0.0038	0.0117	92.00% (0.1287)	0.7718	0.0040	0.0105	97.00% (0.1359)	0.8626	0.0047	0.0039	91.00% (0.0801)
	5	0.8251	0.0013	0.0091	92.00% (0.1178)	0.8005	0.0032	0.0112	93.50% (0.1255)	0.7667	−0.0011	0.0135	94.50% (0.1493)	0.8625	0.0046	0.0035	94.00% (0.0763)
1.00	1	0.8234	−0.0004	0.0021	92.50% (0.0541)	0.7959	−0.0014	0.0020	93.50% (0.0591)	0.7679	0.0001	0.0022	95.00% (0.0587)	0.8600	0.0021	0.0010	92.50% (0.0378)
	2	0.8227	−0.0011	0.0032	92.50% (0.0652)	0.7958	−0.0015	0.0032	93.50% (0.0703)	0.7660	−0.0018	0.0034	96.00% (0.0746)	0.8600	0.0021	0.0013	94.00% (0.0445)
	5	0.8239	0.0001	0.0054	89.50% (0.0871)	0.7971	−0.0002	0.0063	93.00% (0.0921)	0.7674	−0.0004	0.0078	93.00% (0.1079)	0.8605	0.0026	0.0019	94.00% (0.0559)

*Note*: Entries of Var are multiplied 10 for better presentation.p is the prevalence,$\delta_{0}$ and$\delta_{1}$ are specificity and sensitivity,J is the group size, and B‐1, B‐2, B‐3 stand for the individual biomarker 1, 2, 3, respectively.

Moreover, we tested the smaller sample sizeN=10000 with larger prevalencep=0.05,0.1, and keep the remaining settings, see the result in Appendix A of the Supporting Information.

In summary, the simulations demonstrate these findings: (1) the proposed approach of using multiple biomarkers can improve both accuracy and statistical efficiency in the AUC estimates, compared to those of using a single biomarker, (2) as misclassification rates decrease, the proposed estimator becomes more efficient; and (3) when the test is subject to misclassification, the AUC estimates based on group testing can be superior (in both accuracy and efficiency) to those based on individual testing.

Besides that, we added a new simulation where the sensitivity and specificity are mis‐specified and have reported the corresponding results in Appendix B of Supporting Information. In addition, the findings are similar in the scenario of *random individual testing* where the number of groupsn is fixed at 8000 (for bothJ=1 andJ=2,5). We presented the result in Appendix C of Supporting Information.

## 5 Applications

### 5.1 COVID‐19 Detection

We first applied the proposed approach to detecting COVID‐19 with data from the UK Biobank, an international health resource enabling research into the genetic and lifestyle determinants of common diseases. Over 500 000 participants (aged 50–81 on March 16, 2020) from the UK general population were recruited between 2006 and 2010 (aged 40–69), see Satter et al. [37]. Data were available for the period Feburary 25, 2021 to July 12, 2021. Blood samples and symptoms information of the participants were collected and analyzed in this study. In our analysis, we used the self‐antibody test from the UK Biobank. This test recruited UK Biobank participants to perform a SARS‐CoV‐2 antibody self‐test using a lateral flow device (Fortress Fast COVID‐19 device) at home and to report their result to UK Biobank. We focused on participants whose disease statuses are obtained using IgM (https://biobank.ndph.ox.ac.uk/showcase/ukb/docs/c19_antibody_p1_overview.pdf).

Total bilirubin (TBIL), aspartate aminotransferase (AST), and alanine aminotransferase (ALT) levels are three common clinical measurements which are often used in the studies of COVID‐19, see Liu et al. [38], Ali [39], and Kasapoglu et al. [40]. For illustration, we used them as the biomarkers for COVID‐19 infection. While the UK Biobank did not employ group testing to detect COVID‐19, we presented a hypothetical scenario using group‐tested data. This approach is justifiable because the self‐antibody test and the three biomarkers relied on different specimens. We simulated group‐tested outcomes to determine the presence of COVID‐19 independently. To achieve this, we considered the testing results in the dataset as the true disease statuses of the subjects and randomly assigned the self‐antibody test specimens to groups of sizeK. The values of the three biomarkers for each subject remained unchanged.

To apply our proposed method, we first transformed the data to normality using the Box‐Cox transformation. After removing the missing values of IgM, TBIL, ALT, and AST,N=11,837 independent observations of${\left(𝒳,K\right)}^{T}$ were included in the final analysis, out of which 548 subjects tested positive for IgM in the self‐antibody test. To investigate the diagnostic ability of the three biomarkers (TBIL, ALT, and AST), we estimated the ROC curve of their best linear combination, which required the distribution of the combination in the non‐infected and infected population. For comparison, we also calculated the AUC estimator of the best linear combination of the three biomarkers based on individual‐tested results. Based on the UK Biobank data, we set the sensitivity and specificity of the IgM test to be$\delta_{1}=0.952$ and$\delta_{0}=0.960$ (see https://biobank.ndph.ox.ac.uk/showcase/refer.cgi?id=4513), and assumed that$\delta_{1}^{*}(J,d)=\delta_{1}d/\{d+\lambda (J-d)\}$ withλ=0.02.

**FIGURE 1:**
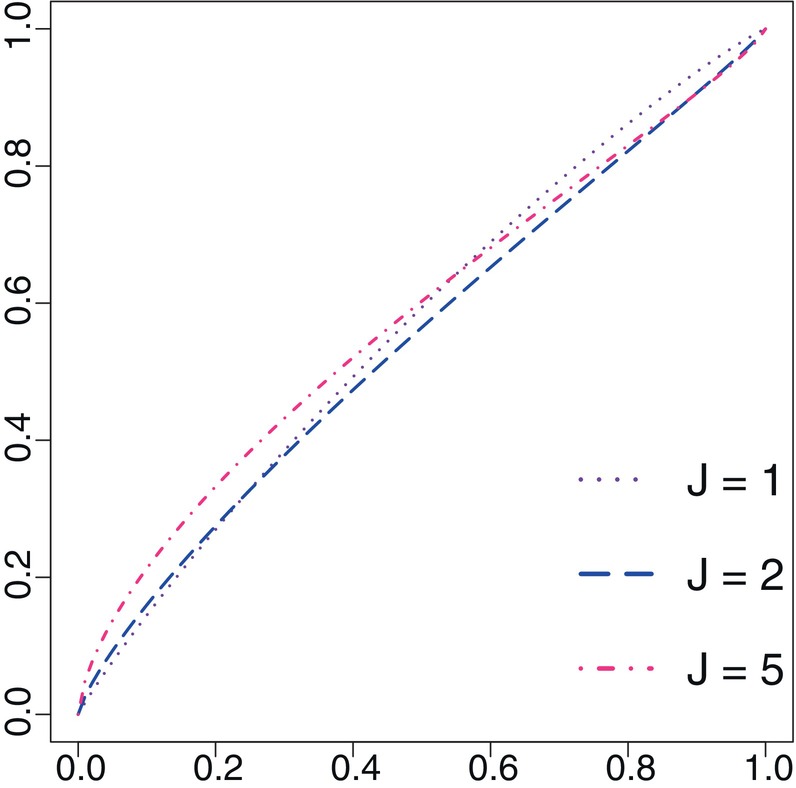
ROC curve estimates of the best linear combination of the three biomarkers (TBIL, ALT, and AST) for the group sizes ofJ=1,2,5, using data from UK Biobank.

Table 3 presents the AUC estimators of the three individual biomarkers and their best linear combination based on the group‐tested results and individual‐tested results. The variance of the AUC estimator is calculated using 1000 bootstrap replicates. From the table, it can be found that the AUC estimator of the best combination is always larger than that of individual biomarkers. Among the group sizes considered, the best efficiency is attained by the AUC estimator underJ=2. The ROC curve of the best linear combination of the three biomarkers is displayed in Figure 1.

**TABLE 3:** Analysis of the UK Biobank COVID‐19 data: estimates (Est) and variances (Var) of AUC for the three individual biomarker (TBIL, ALT, and AST) and their best linear combination based on individual (J=1) and group testing (J=2,5) approaches.

	Individual						Combined		
	TBIL		ALT		AST				
J	Est	Var	Est	Var	Est	Var	Est	Var	95% CI
1	0.5318	0.0029	0.5213	0.0021	0.5218	0.0022	0.5730	0.0057	(0.5264, 0.6196)
2	0.5189	0.0021	0.5178	0.0017	0.5209	0.0021	0.5616	0.0041	(0.5217, 0.6015)
5	0.5278	0.0043	0.5316	0.0044	0.5260	0.0034	0.5914	0.0079	(0.5362, 0.6467)

*Note*: Entries of Var are multiplied 10 for better presentation.J is the size of each group.

### 5.2 Chlamydia Detection

Another real data analysis concerns genital chlamydia infections and is conducted data from the National Health and Nutrition Examination Survey (NHANES; https://www.cdc.gov/nchs/nhanes/index.htm), a large‐scale population study aimed at evaluating the health and nutritional well‐being of individuals across the United States.

The NHANES study collected urine samples from participants aged 18–39 and tests for genital chlamydia infections using the DNA strand displacement amplification method. The publicly released data includes assay results of eligible participants. Chlamydia, caused by *Chlamydia trachomatis*, is a common sexually transmitted disease that can affect the levels of monocyte, neutrophils, and albumin, as reported in Datta et al. [41], Naglak, Morrison, and Morrisons [42], and Stoke and Isada [43]. In this analysis, we considered using monocyte, neutrophils, and albumin as biomarkers for chlamydia infections.

We collected data on chlamydia and three biomarkers including monocyte, neutrophils, and albumin from six consecutive and independent surveys of NHANES (1999–2000, 2001–2002, 2003–2004, 2005–2006, 2007–2008, 2009–2010). To address the potential impact of oversampling and the complex survey design, we performed resampling on the data from each two‐year survey dataset. This resampling was done with replacement, using sampling weights proportional to the probabilities, while keeping the sample size consistent with the original dataset. Subsequently, we combined these resampled datasets to create a large sample.

After excluding subjects with missing values for chlamydia, monocyte, neutrophils, and albumin, our final working dataset consisted ofN=12424 independent observations of${\left(𝒳,K\right)}^{T}$. Among these observations, 221 subjects tested positive for chlamydia. Similar to the UK Biobank data, the NHANES study did not utilize group testing to detect chlamydia. Since the detection of chlamydia infections and the measurement of monocyte, neutrophils, and albumin used different specimens, we generated group‐tested outcomes for disease presence independently. We achieved this by considering the testing results available in the current dataset. Similarly, we use the Box‐Cox transformation to transform the original data of monocyte, neutrophils, and albumin. We estimated the ROC curve for the optimal linear combination of the three biomarkers using a process similar to the COVID‐19 example, except that we assumed a specificity of$\delta_{0}=0.99$ and a sensitivity of$\delta_{1}=0.9$, and assumed that$\delta_{1}^{*}(J,d)=\delta_{1}d/\{d+\lambda (J-d)\}$ withλ=0.02.

**FIGURE 2:**
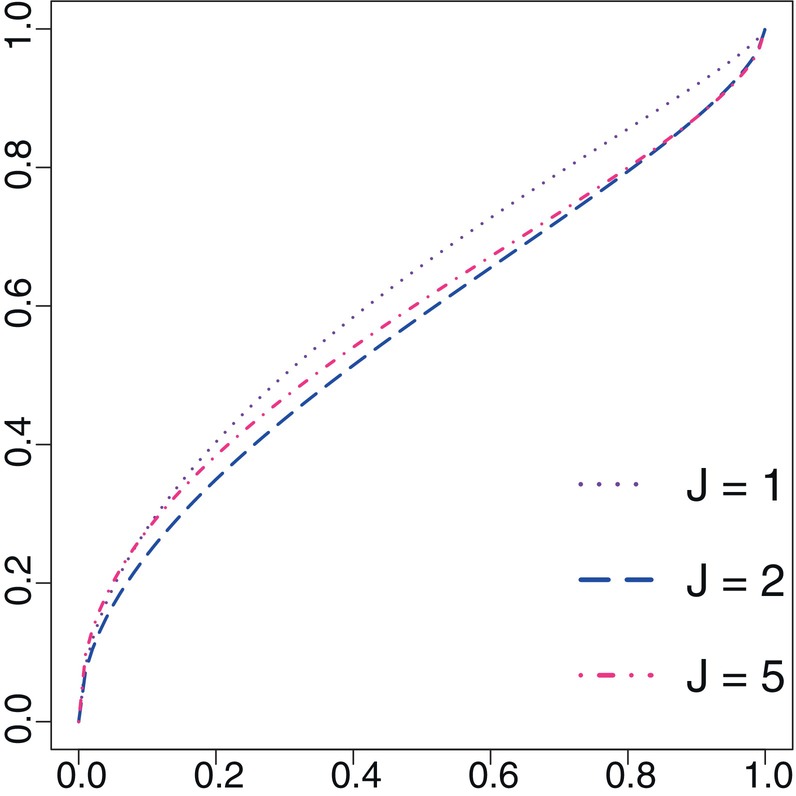
ROC curve estimates of the best linear combination of the three biomarkers (Monocyte, Neutrophils, and Albumin) for the group sizes ofJ=1,2,5, using data from the NHANES study.

The AUC estimators for individual biomarkers and their best linear combination, based on the group‐tested and individual‐tested results, are presented in Table 4. The variance of the AUC estimator is computed using 1000 bootstrap replicates. The table reveals that the AUC estimates for the best combination consistently outperform those for individual biomarkers, with an average increase of 10.31%. The AUC estimator achieves the best efficiency whenJ=2, with a relative efficiency of 0.92 compared to the individual‐tested results (J=1). The ROC curve of the best linear combination of the three biomarkers is displayed in Figure 2.

**TABLE 4:** Analysis of the chlamydia data: estimates (Est) and variances (Var) of AUC for the three individual biomarker (monocyte, neutrophils and albumin) and their best linear combination based on individual (J=1) and group testing (J=2,5) approaches.

	Individual						Combined		
	Monocyte		Neutrophils		Albumin				
J	Est	Var	Est	Var	Est	Var	Est	Var	95% CI
1	0.5333	0.0052	0.5734	0.0191	0.5697	0.0076	0.6345	0.0076	(0.5804, 0.6885)
2	0.5267	0.0039	0.5594	0.0135	0.5293	0.0054	0.5859	0.0070	(0.5339, 0.6379)
5	0.5340	0.0070	0.5957	0.0133	0.5490	0.0102	0.6051	0.0089	(0.5466, 0.6636)

*Note*: Entries of Var are multiplied 10 for better presentation.J is the size of each group.

## 6 Discussion

In this article, we consider the problem of combining multiple biomarkers to improve the diagnostic accuracy using AUC and ROC curve as a criterion, when only group‐based test results on the disease status are available while biomarker value at individual level can be observed.

To ease computation, we proposed a pairwise model fitting approach to estimate the conditional distributions of the optimal linear combination of the multiple biomarkers given the disease status and its diagnostic accuracy. This approach is advantageous whenever fitting the full multivariate model is not possible or too time‐consuming. Extensive simulations show that the proposed estimators based on group testing can improve statistical efficiency over the individual testing in the presence of misclassification.

Here, we focus on normal distributions since obtaining the optimal linear combination and its corresponding AUC is challenging for a general multivariate distribution even the most common ones. This is because even for the most common multivariate distributions, it is very difficult to derive the distribution of a linear combination of the individual variate. The multivariate normal distributions are the only exception since it is well known that a linear combination of their variates is also normal, thus warrants an explicit form of the optimal linear combination and its AUC. It is worth pointing out that treating non‐normal biomarkers as normal biomarkers may lead to substantial bias, see the results in Appendix D of Supporting Information.

In addition to the measures discussed in this article, alternative summary measures of a biomarker's diagnostic capability, such as the Youden index, offer a different perspective. The Youden index, represented as$J=\max_{c}\text{Sensitivity}(c)+\text{Specificity}(c)-1$, quantifies the maximum potential diagnostic effectiveness of a biomarker by assigning equal importance to sensitivity and specificity. Exploring the extension of the findings presented in this article to other diagnostic measures warrants further investigation and research.

We assumed that the misclassification of the single and pooled assay for the disease outcome is known. It is worth noting that mis‐specifying the sensitivity and specificity may yield poor estimation. Haber et al. [28] showed the importance of estimating these misclassification processes in the population under study. See also sensitivity analysis results in Appendix B of the Supporting Information. Future research will focus on the efficient design of a validation study to estimate these processes.

Cautions need to be taken that when the prevalence is low and the sample size is small, resulting in a relatively small number of groups tested positive, the confidence intervals may have coverage below the expected level and may not be reliable.

Our work focuses on the Master pool testing. Other group testing protocols have been developed in the literature, such as array testing and master pool testing with retesting. With more data available from further testing, we expect that more efficient estimation for the ROC and its AUC can be obtained. Extending our method to other types of group testing data appears to be technically more challenging, and warrants further investigation.

Finally, if measuring the biomarkers' levels is costly or time consuming, one could also consider pooling biospecimen to measure the biomarkers to save cost and time. In this case, individual level is not observed on the biomarkers, but rather one observes an average of a biomarker's levels from individuals in the pool. This additional feature in the data structure creates more technical difficulty in deriving the likelihood, the ROC and its AUC. Future research is warranted in this direction.

## Conflicts of Interest

The authors declare no conflicts of interest.

## Supporting information




**Data S1.** Supporting Information

## Data Availability

The COVID‐19 data that support the findings of this study are available from UK Biobank. Restrictions apply to the availability of these data, which were used under the license for this study. Data are available at https://www.ukbiobank.ac.uk/ with the permission of UK Biobank. The chlamydia data that support the findings of this study are available in National Health and Nutrition Examination Survey (NHANES) at https://www.cdc.gov/nchs/nhanes/index.htm.
