# Supplementary material for: Combining Biomarkers to Improve Diagnostic Accuracy in Detecting Diseases With Group‐Tested Data
Source: Stat Med. 2024 Oct 7;43(27):5182–92. doi: 10.1002/sim.10230 (PMC11583953; doi:10.1002/sim.10230)
Supplement: Supplementary file 1 — Data S1. Supporting Information [file SIM-43-5182-s001.pdf]

Received <day> <Month>, <year>; | Revised <day> <Month>, <year>; | Accepted <day> <Month>, <year>

DOI: xxx/xxxx

ARTICLE TYPE

Supplementary material of “Combining Biomarkers to Improve Diagnostic Accuracy in Detecting Diseases with Group-tested Data”

Jin Yang[†1] | Wei Zhang[†2] | Paul S. Albert[3] | Aiyi Liu[1] | Zhen Chen*[1]

[1]Biostatistics and Bioinformatics Branch, *Eunice Kennedy Shriver* National Institute of Child Health and Human Development, National Institutes of Health, Bethesda, MD 20817
[2]Academy of Mathematics and Systems Science, Chinese Academy of Sciences, Beijing, China 100190
[3]Biostatistics Branch, Division of Cancer Epidemiology and Genetics, National Cancer Institute, National Institutes of Health, Bethesda, MD 20892

Correspondence
*Zhen Chen, Biostatistics and Bioinformatics Branch, *Eunice Kennedy Shriver* National Institute of Child Health and Human Development, National Institutes of Health, Bethesda, MD 20817
Email: chenzhe@mail.nih.gov

Abstract

We consider the problem of combining multiple biomarkers to improve the diagnostic accuracy of detecting a disease when only group-tested data on the disease status is available. There are several challenges in addressing this problem, including unavailable individual disease statuses, differential misclassification depending on group size and number of diseased individuals in the group, and extensive computation due to a large number of possible combinations of multiple biomarkers. To tackle this issue, we propose a pairwise model fitting approach to estimating the distribution of the optimal linear combination of biomarkers and its diagnostic accuracy under the assumption of a multivariate normal distribution. The approach is evaluated in simulation studies and applied to data on chlamydia detection and COVID-19 diagnosis.

KEYWORDS:
AUC; Differential misclassification; Joint model; Multiple biomarkers

1 | APPENDIX A

Tables 1 and 2 present the estimators of the prevalence and AUCs with higher prevalence $p = 0.05, 0.1$ and smaller sample size $N = 10000$, respectively. As expected, combining biomarker (“Combined") yields larger AUC estimates, indicating improved diagnostic accuracy compared to individual biomarkers (“Individual"). Similar to the findings for the prevalence estimator, as the misclassification error decreases, the variance of the estimators decreases. Furthermore, the AUC estimator of the combined biomarkers is always more efficient than their single biomarker counterparts, as its variance is smaller than all those in the individual setting. Moreover, the AUC estimates based on group testing are close to that based on individual testing. When misclassification exists ($\delta_0,\ \delta_1 < 1.00$), there is always an estimator based on group testing that yields smaller variance than the individual testing counterpart. However, you could find that when $p = 0.1$, the relative efficiency of the combined biomarkers is larger than 1, which means the variance of the estimator with $K = 2, 5$ is larger than that with $K = 1$. Biomarker 3 also showed the same trend. This is not unexpected since when we fixed the other parameters, i.e. π_0, π_1, N and n, then the group testing is more efficient than the individual testing when the prevalence is relatively low, see Liu et al. (2012)[1].

Table 1 insert here.

[†]Jin Yang and Wei Zhang are the co-first authors.

Table 2 insert here.

2 | APPENDIX B

We have also presented a new simulation where the sensitivity and specificity are mis-specified and have reported the corresponding results in Table 3. In this scenario, sample size $N = 15000$, the prevalence $p = 0.02$ and the original sensitivity and specificity $\delta_1 = \delta_0 = 0.85$. It is clear to see that the estimates have large bias when we mis-specify the sensitivity and specificity. We also added the mean squared error (MSE) in the table. It is easy to find that the estimators of prevalence and AUC have larger MSE when the sensitivity and specificity are mis-specified. For example, when $\delta_1 = \delta_0 = 0.85$, the MSE of combined AUC at $J = 2$ is 0.0088 which is smaller than 0.4124 when $\delta_1 = \delta_0 = 0.95$. Same conclusions can be drawn from the results of the prevalence. Thus it is important that the sensitivity and specificity be correctly specified for the binary test of the disease. In practice, we recommend that a small validation study be performed to estimate sensitivity and specificity when it is not well established in the population under consideration.

Table 3 insert here.

3 | APPENDIX C

Tables 4 and 5 present the estimators of the prevalence and AUCs in the scenario of *random individual testing*, respectively. Here, the number of groups n is fixed at 8000. From Table 4, one can see that the variance of the prevalence estimator decreases as the misclassification error decreases. Likewise, as the group size increases, the efficiency of the prevalence estimator increases (i.e. smaller variance). Table 5 shows that the variance of the AUC estimator decreases as the prevalence increases or the misclassification error decreases. Among all group sizes considered, the best efficiency is usually achieved by the AUC estimator with $J = 5$. For example, when $p = 0.02$ and $\delta_0 = \delta_1 = 0.95$, the variance of combined AUC at $J = 5$ is the smallest among all choices of group size J. Compared to *full individual testing*, CPs under *random individual testing* in Tables 4 and 5 are slightly lower than the nominal level. This is not unexpected, under the case of the smaller sample size and low prevalence. We observed improved CPs when the prevalence increases from 0.01 to 0.02 in these two tables.

Table 4 insert here.

Table 5 insert here.

4 | APPENDIX D

Tables 6 presents the biases of the AUCs when the data generated from multivariate log-normal distribution & gamma distribution. Here the sample size is $N = 15000, 20000$, with the prevalence $p = 0.02, 0.05$. The true value of AUC of Log-normal and gamma was approximated by Mann-Whitney U statistics with 20000 sample data point.

In Log-normal setting, for the subjects with $D = 0$, values of biomarkers were generated from the multivariate log-normal distribution with mean $(0, 0, 0)$ and covariance matrix with diagonal elements 1, 1, and 1 and pairwise correlation coefficients 1/3, 1/3, and 1/3; otherwise, they were generated from the multivariate log-normal distribution with mean $(1.6, 1.5, 1.2)$ and covariance matrix with diagonal elements 1.4^2, 1.5^2, and 1.3^2 and pairwise correlation coefficients 0.5, 0.6, and 0.3, respectively. In gamma setting, for the subjects with $D = 0$, values of biomarkers were generated from the multivariate gamma distribution with shape $(1, 2, 1.5)$, rate $(1.3, 1.2, 2.3)$ and correlation structure with diagonal elements 1, 1 and 1, and pairwise correlation coefficients 0.3, 0.3 and 0.3; otherwise, they were generated from the multivariate gamma distribution with shape $(1.2, 1.1, 1)$,

rate (1.4, 1.6, 2.5) and correlation structure with diagonal elements 1, 1 and 1, and pairwise correlation coefficients 0.4, 0.7 and 0.2. The true AUC of Log-normal and Gamma are 0.8636 and 0.8290, respectively.

It is easy to see that the biases of the proposed AUC estimate is large when the data generated from multivariate log-normal or gamma distributions. For example, when $N = 15000$, $\delta_0 = \delta_1 = 0.95$ and $p = 0.05$, the bias and absolute of relative error at $J = 2$ are -0.0884 and 10.23% from Log-normal data, and are 0.0407 and 4.92% from gamma data. In summary, it is not appropriate to use the proposed approach when data generated from non-normal distributions.

Table 6 insert here.

References

1. Liu C, Liu A, Zhang Z, Albert PS. Optimality of group testing in the presence of misclassification. *Biometrika* 2012; 99(1): 245-251.

□

TABLE 1 Simulation results for the prevalence estimator based on the group and *full individual testing* approaches: estimate (Est), bias (Bias), variance (Var), coverage probability (CP) and average confidence interval length (ACIL) of the estimators for the individual (B-1, B-2, B-3) and combined biomarkers. Entries of Est and Bias are multiplied by 100, and entries of Var are multiplied by 10000 for better presentation. p is the prevalence, δ_0 and δ_1 are the specificity and sensitivity, J is the group size, and B-1, B-2, B-3 stand for individual biomarker 1, 2, 3, respectively.

		$p = 0.05$															
		Individual												Combined			
		B-1				B-2				B-3							
$\delta_0 = \delta_1$	J	Est	Bias	Var	CP(ACIL)	Est	Bias	Var	CP(ACIL)	Est	Bias	Var	CP(ACIL)	Est	Bias	Var	CP(ACIL)
0.90	1	5.0223	0.0223	0.1435	98.00%(0.0167)	5.0113	0.0113	0.1371	98.00%(0.0166)	5.0253	0.0253	0.1500	96.50%(0.0169)	5.0105	0.0105	0.0817	97.50%(0.0130)
	2	5.0264	0.0264	0.1165	96.00%(0.0141)	5.0281	0.0281	0.1136	96.00%(0.0140)	5.0289	0.0289	0.1227	96.50%(0.0142)	5.0112	0.0112	0.0871	96.50%(0.0121)
	5	5.0190	0.0190	0.0881	97.00%(0.0127)	5.0132	0.0132	0.0856	96.50%(0.0127)	5.0208	0.0208	0.0910	96.00%(0.0128)	5.0051	0.0051	0.0756	96.00%(0.0115)
0.95	1	5.0308	0.0308	0.1118	94.50%(0.0126)	5.0272	0.0272	0.1080	94.00%(0.0125)	5.0373	0.0373	0.1134	94.50%(0.0127)	5.0220	0.0220	0.0817	94.50%(0.0108)
	2	5.0311	0.0311	0.0827	94.00%(0.0113)	5.0342	0.0342	0.0807	93.50%(0.0113)	5.0335	0.0335	0.0823	94.00%(0.0114)	5.0284	0.0284	0.0676	94.50%(0.0103)
	5	5.0237	0.0237	0.0721	93.75%(0.0109)	5.0221	0.0221	0.0724	95.31%(0.0109)	5.0236	0.0236	0.0740	93.75%(0.0110)	5.0178	0.0178	0.0657	93.23%(0.0102)
1.00	1	5.0024	0.0024	0.0491	94.00%(0.0085)	5.0018	0.0018	0.0491	94.00%(0.0085)	5.0016	0.0015	0.0489	94.00%(0.0085)	5.0149	0.0149	0.0460	95.00%(0.0082)
	2	5.0032	0.0032	0.0529	93.00%(0.0087)	5.0045	0.0045	0.0537	93.00%(0.0087)	5.0029	0.0029	0.0534	93.00%(0.0087)	5.0161	0.0161	0.0519	93.00%(0.0084)
	5	4.9967	-0.0033	0.0638	93.00%(0.0093)	4.9950	-0.0050	0.0652	91.50%(0.0093)	4.9951	-0.0049	0.0663	91.50%(0.0093)	5.0042	0.0042	0.0601	92.00%(0.0089)

		$p = 0.1$															
		Individual												Combined			
		B-1				B-2				B-3							
$\delta_0 = \delta_1$	J	Est	Bias	Var	CP(ACIL)	Est	Bias	Var	CP(ACIL)	Est	Bias	Var	CP(ACIL)	Est	Bias	Var	CP(ACIL)
0.90	1	10.0337	0.0337	0.2000	96.50%(0.0186)	10.0303	0.0303	0.1924	98.00%(0.0184)	10.0343	0.0343	0.2047	97.00%(0.0188)	10.0013	0.0013	0.1497	94.00%(0.0156)
	2	10.0415	0.0415	0.1914	96.00%(0.0168)	10.0410	0.0410	0.1929	96.50%(0.0168)	10.0411	0.0411	0.2019	96.00%(0.0169)	10.0229	0.0229	0.1599	93.50%(0.0150)
	5	10.0212	0.0212	0.2063	94.00%(0.0171)	10.0064	0.0064	0.2052	94.00%(0.0170)	10.0179	0.0179	0.2081	94.50%(0.0173)	9.9826	-0.0174	0.1649	95.50%(0.0153)
0.95	1	10.0384	0.0384	0.1689	94.00%(0.0150)	10.0397	0.0397	0.1621	94.50%(0.0150)	10.0414	0.0414	0.1700	94.00%(0.0151)	10.0328	0.0328	0.1365	95.00%(0.0135)
	2	10.0444	0.0444	0.1477	93.50%(0.0143)	10.0421	0.0421	0.1470	93.00%(0.0143)	10.0466	0.0466	0.1492	93.00%(0.0143)	10.0423	0.0423	0.1277	92.00%(0.0132)
	5	10.0365	0.0365	0.1638	92.50%(0.0151)	10.0432	0.0432	0.1650	93.00%(0.0151)	10.0342	0.0342	0.1702	93.00%(0.0153)	10.0231	0.0231	0.1441	91.50%(0.0139)
1.00	1	10.0057	0.0057	0.0967	94.50%(0.0117)	10.0056	0.0056	0.0969	93.50%(0.0117)	10.0065	0.0065	0.0963	94.50%(0.0117)	10.0252	0.0252	0.0851	94.00%(0.0110)
	2	10.0012	0.0012	0.1082	94.00%(0.0121)	10.0027	0.0027	0.1091	94.50%(0.0121)	10.0027	0.0027	0.1096	93.50%(0.0121)	10.0145	0.0145	0.0998	93.50%(0.0113)
	5	9.9951	-0.0049	0.1306	94.00%(0.0135)	9.9909	-0.0091	0.1338	94.00%(0.0136)	9.9975	-0.0025	0.1339	93.50%(0.0137)	10.0012	0.0012	0.1186	94.50%(0.0126)

TABLE 2 Simulation results for the AUC estimator based on the group and *full individual testing* approaches: estimate (Est), bias (Bias) and variance (Var), coverage probability (CP) and average confidence interval length (ACIL) of the estimators for the individual (B-1, B-2, B-3) and combined biomarkers. Entries of Var are multiplied 10 for better presentation. p is the prevalence, δ_0 and δ_1 are specificity and sensitivity, J is the group size, and B-1, B-2, B-3 stand for the individual biomarker 1, 2, 3, respectively.

		$p = 0.05$															
		Individual												Combined			
		B-1				B-2				B-3							
$\delta_0 = \delta_1$	J	Est	Bias	Var	CP(ACIL)	Est	Bias	Var	CP(ACIL)	Est	Bias	Var	CP(ACIL)	Est	Bias	Var	CP(ACIL)
0.90	1	0.8209	-0.0029	0.0066	95.50%(0.1062)	0.7980	0.0007	0.0075	94.00%(0.1072)	0.7705	0.0027	0.0081	94.00%(0.1093)	0.8591	0.0012	0.0023	96.00%(0.0634)
	2	0.8203	-0.0035	0.0060	93.00%(0.0964)	0.7983	0.0010	0.0054	95.00%(0.0988)	0.7685	0.0007	0.0055	95.50%(0.1075)	0.8586	0.0007	0.0019	96.00%(0.0602)
	5	0.8234	-0.0004	0.0064	91.50%(0.0980)	0.7992	0.0019	0.0070	93.00%(0.1026)	0.7657	-0.0021	0.0094	93.00%(0.1215)	0.8578	-0.0001	0.0024	93.00%(0.0606)
0.95	1	0.8229	-0.0009	0.0042	92.00%(0.0782)	0.7953	-0.0020	0.0043	92.00%(0.0800)	0.7673	-0.0005	0.0042	95.00%(0.0816)	0.8582	0.0003	0.0018	94.00%(0.0509)
	2	0.8227	-0.0011	0.0032	95.00%(0.0771)	0.7948	-0.0025	0.0036	97.00%(0.0800)	0.7677	-0.0001	0.0043	96.00%(0.0853)	0.8575	-0.0004	0.0015	94.00%(0.0495)
	5	0.8231	-0.0007	0.0049	94.79%(0.0856)	0.7970	-0.0003	0.0059	92.19%(0.0907)	0.7662	-0.0016	0.0071	94.27%(0.1044)	0.8570	-0.0009	0.0021	94.27%(0.0535)
1.00	1	0.8250	0.0012	0.0010	95.50%(0.0418)	0.7983	0.0010	0.0013	93.50%(0.0461)	0.7669	-0.0009	0.0013	96.00%(0.0464)	0.8584	0.0005	0.0006	95.50%(0.0297)
	2	0.8224	-0.0014	0.0018	92.50%(0.0518)	0.7969	-0.0004	0.0022	93.50%(0.0558)	0.7680	0.0002	0.0023	94.00%(0.0587)	0.8586	0.0007	0.0008	95.00%(0.0344)
	5	0.8227	-0.0011	0.0035	92.50%(0.0711)	0.7981	0.0008	0.0040	92.00%(0.0769)	0.7665	-0.0013	0.0051	94.00%(0.0888)	0.8583	0.0004	0.0013	93.00%(0.0445)

		$p = 0.1$															
		Individual												Combined			
		B-1				B-2				B-3							
$\delta_0 = \delta_1$	J	Est	Bias	Var	CP(ACIL)	Est	Bias	Var	CP(ACIL)	Est	Bias	Var	CP(ACIL)	Est	Bias	Var	CP(ACIL)
0.90	1	0.8236	-0.0002	0.0022	96.50%(0.0612)	0.7970	-0.0003	0.0020	96.00%(0.0601)	0.7697	0.0019	0.0025	95.00%(0.0630)	0.8574	-0.0005	0.0007	98.00%(0.0365)
	2	0.8229	-0.0009	0.0019	97.00%(0.0592)	0.7976	0.0003	0.0019	95.50%(0.0608)	0.7690	0.0012	0.0027	95.50%(0.0652)	0.8570	-0.0009	0.0008	95.50%(0.0357)
	5	0.8257	0.0019	0.0029	95.50%(0.0679)	0.7956	-0.0017	0.0031	97.00%(0.0718)	0.7690	0.0012	0.0053	94.50%(0.0859)	0.8577	-0.0002	0.0010	94.00%(0.0390)
0.95	1	0.8222	-0.0016	0.0016	93.50%(0.0470)	0.7967	-0.0006	0.0016	91.00%(0.0476)	0.7654	-0.0024	0.0012	97.50%(0.0490)	0.8571	-0.0008	0.0007	93.50%(0.0300)
	2	0.8222	-0.0016	0.0014	96.00%(0.0488)	0.7959	-0.0014	0.0014	97.50%(0.0506)	0.7668	-0.0010	0.0020	94.50%(0.0546)	0.8571	-0.0008	0.0007	92.00%(0.0305)
	5	0.8239	0.0001	0.0027	92.00%(0.0613)	0.7976	0.0003	0.0022	96.00%(0.0649)	0.7670	-0.0008	0.0037	94.50%(0.0764)	0.8578	-0.0001	0.0009	90.50%(0.0359)
1.00	1	0.8246	0.0008	0.0006	95.50%(0.0301)	0.7976	0.0003	0.0006	97.00%(0.0330)	0.7677	-0.0001	0.0007	95.50%(0.0333)	0.8580	0.0001	0.0003	94.50%(0.0216)
	2	0.8246	0.0008	0.0008	96.00%(0.0374)	0.7967	-0.0006	0.0012	94.50%(0.0411)	0.7689	0.0011	0.0012	94.00%(0.0431)	0.8582	0.0003	0.0004	94.00%(0.0249)
	5	0.8242	0.0004	0.0020	93.50%(0.0543)	0.7973	0.0000	0.0023	92.50%(0.0593)	0.7705	0.0027	0.0027	93.50%(0.0664)	0.8576	-0.0003	0.0007	94.00%(0.0326)

TABLE 3 Simulation results for the prevalence and AUC based on the group and *full individual testing* approaches: estimate (Est), bias (Bias), mean squared error (MSE), variance (Var), coverage probability (CP) and average confidence interval length (ACIL) of the estimators for the individual (B-1, B-2, B-3) and combined biomarkers. In prevalence results, Entries of Est and Bias are multiplied by 100, and entries of Var and MSE are multiplied by 10000 for better presentation. In AUC results, and entries of Var and MSE are multiplied by 10 for better presentation. p is the prevalence, δ_0 and δ_1 are the specificity and sensitivity, J is the group size, and B-1, B-2, B-3 stand for individual biomarker 1, 2, 3, respectively. The true sensitivity and specificity that generated the simulation data were both 0.85; The corresponding values in the first column were used to fit the models.

		$p = 0.02$																			
		Individual (prevalence)															Combined (prevalence)				
		B-1					B-2					B-3									
$\delta_0 = \delta_1$	J	Est	Bias	Var	MSE	CP(ACIL)	Est	Bias	Var	MSE	CP(ACIL)	Est	Bias	Var	MSE	CP(ACIL)	Est	Bias	Var	MSE	CP(ACIL)
	1	2.0035	0.0035	0.1522	0.1522	92.00%(0.0152)	1.9859	-0.0141	0.1450	0.1452	94.50%(0.0154)	1.9916	-0.0084	0.1688	0.1688	93.00%(0.0166)	1.9924	-0.0076	0.0555	0.0555	95.50%(0.0099)
0.85	2	1.9648	-0.0352	0.0919	0.0931	96.50%(0.0127)	1.9757	-0.0243	0.1036	0.1042	94.00%(0.0124)	1.9759	-0.0241	0.0991	0.0996	97.00%(0.0130)	1.9833	-0.0167	0.0510	0.0512	95.50%(0.0092)
	5	1.9852	-0.0148	0.0642	0.0645	93.91%(0.0099)	1.9800	-0.0200	0.0603	0.0607	94.42%(0.0098)	1.9842	-0.0158	0.0620	0.0623	94.42%(0.0099)	1.9929	-0.0071	0.0411	0.0411	94.92%(0.0082)
	1	14.1548	12.1548	0.2351	147.9748	0.00%(0.0132)	14.1472	12.1472	0.2440	147.7994	0.00%(0.0132)	14.2253	12.2253	0.1277	149.5862	0.00%(0.0132)	12.7156	10.7156	0.5515	115.3760	0.00%(0.0181)
0.95	2	8.3633	6.3633	0.1043	40.5961	0.00%(0.0106)	8.3475	6.3475	0.1225	40.4136	0.00%(0.0106)	8.4153	6.4153	0.0856	41.2414	0.00%(0.0106)	7.7124	5.7124	0.2062	32.8374	0.00%(0.0112)
	5	4.7630	2.7630	0.0587	7.6929	0.00%(0.0083)	4.7440	2.7440	0.0575	7.5870	0.00%(0.0083)	4.7728	2.7728	0.0581	7.7467	0.00%(0.0083)	4.3674	2.3674	0.0580	5.6624	0.00%(0.0081)

		$p = 0.02$																			
		Individual (AUC)															Combined (AUC)				
		B-1					B-2					B-3									
$\delta_0 = \delta_1$	J	Est	Bias	Var	MSE	CP(ACIL)	Est	Bias	Var	MSE	CP(ACIL)	Est	Bias	Var	MSE	CP(ACIL)	Est	Bias	Var	MSE	CP(ACIL)
	1	0.8255	0.0017	0.0291	0.0291	93.00%(0.2115)	0.8054	0.0081	0.0348	0.0354	88.00%(0.2201)	0.7742	0.0064	0.0449	0.0453	91.00%(0.2478)	0.8671	0.0092	0.0078	0.0086	90.50%(0.1146)
0.85	2	0.8282	0.0044	0.0239	0.0241	93.50%(0.1950)	0.8019	0.0046	0.0278	0.0280	92.00%(0.1927)	0.7807	0.0129	0.0280	0.0296	92.50%(0.2206)	0.8678	0.0099	0.0078	0.0088	89.50%(0.1082)
	5	0.8244	0.0006	0.0192	0.0192	90.36%(0.1770)	0.8029	0.0056	0.0210	0.0213	92.39%(0.1781)	0.7723	0.0045	0.0296	0.0298	92.89%(0.2035)	0.8655	0.0076	0.0068	0.0074	89.88%(0.1017)
	1	0.5619	-0.2619	0.0020	0.6879	0.00%(0.0328)	0.5591	-0.2382	0.0013	0.5688	0.00%(0.0328)	0.5463	-0.2215	0.0009	0.4916	0.00%(0.0333)	0.6193	-0.2386	0.0062	0.5754	0.00%(0.0618)
0.95	2	0.6018	-0.2220	0.0020	0.4951	0.00%(0.0474)	0.5985	-0.1988	0.0027	0.3980	0.00%(0.0473)	0.5805	-0.1873	0.0019	0.3526	0.00%(0.0519)	0.6564	-0.2015	0.0066	0.4124	0.00%(0.0532)
	5	0.6600	-0.1638	0.0031	0.2714	0.00%(0.0737)	0.6534	-0.1439	0.0034	0.2106	0.00%(0.0712)	0.6305	-0.1373	0.0052	0.1937	0.00%(0.0863)	0.7371	-0.1208	0.0094	0.1552	0.00%(0.0667)

TABLE 4 Simulation results for the prevalence estimator based on the group and *random individual testing* approaches: estimate (Est), bias (Bias), variance (Var), coverage probability (CP) and average confidence interval length (ACIL) of the estimators for the individual (B-1, B-2, B-3) and combined biomarkers. Entries of Est and Bias are multiplied by 100, and entries of Var are multiplied by 10000 for better presentation. p is the prevalence, δ_0 and δ_1 are the specificity and sensitivity, J is the group size, and B-1, B-2, B-3 stand for individual biomarker 1, 2, 3, respectively.

		$p = 0.01$															
		Individual												Combined			
		B-1				B-2				B-3							
$\delta_0 = \delta_1$	J	Est	Bias	Var	CP(ACIL)	Est	Bias	Var	CP(ACIL)	Est	Bias	Var	CP(ACIL)	Est	Bias	Var	CP(ACIL)
0.90	1	0.9907	-0.0093	0.1408	92.02%(0.0144)	0.9934	-0.0066	0.1285	88.30%(0.0136)	0.9799	-0.0201	0.1607	91.49%(0.0170)	1.0103	0.0103	0.0479	93.09%(0.0091)
	2	1.0115	0.0115	0.0493	94.97%(0.0087)	1.0105	0.0105	0.0491	94.97%(0.0086)	1.0165	0.0165	0.0515	95.48%(0.0092)	1.0125	0.0125	0.0276	93.97%(0.0064)
	5	0.9939	-0.0061	0.0103	95.98%(0.0042)	0.9934	-0.0066	0.0102	95.98%(0.0042)	0.9933	-0.0067	0.0105	95.48%(0.0043)	0.9928	-0.0072	0.0073	95.98%(0.0035)
0.95	1	0.9991	-0.0009	0.0702	94.82%(0.0105)	0.9852	-0.0148	0.0649	94.82%(0.0105)	0.9865	-0.0135	0.0765	93.26%(0.0112)	1.0139	0.0139	0.0332	93.78%(0.0077)
	2	1.0075	0.0075	0.0267	94.00%(0.0063)	1.0100	0.0100	0.0259	92.50%(0.0062)	1.0082	0.0082	0.0283	93.00%(0.0064)	1.0113	0.0113	0.0174	93.50%(0.0051)
	5	0.9939	-0.0061	0.0065	94.50%(0.0031)	0.9929	-0.0071	0.0066	95.50%(0.0031)	0.9934	-0.0066	0.0067	94.00%(0.0031)	0.9979	-0.0021	0.0053	93.50%(0.0028)
1.00	1	1.0077	0.0077	0.0133	95.00%(0.0044)	1.0076	0.0076	0.0133	95.00%(0.0044)	1.0078	0.0078	0.0133	94.50%(0.0044)	1.0170	0.0170	0.0122	95.50%(0.0042)
	2	1.0055	0.0055	0.0075	95.00%(0.0031)	1.0056	0.0056	0.0075	94.00%(0.0031)	1.0054	0.0054	0.0075	94.00%(0.0031)	1.0129	0.0129	0.0069	94.00%(0.0030)
	5	0.9990	-0.0010	0.0027	95.50%(0.0020)	0.9991	-0.0009	0.0028	94.50%(0.0020)	0.9992	-0.0008	0.0027	96.00%(0.0020)	1.0057	0.0057	0.0025	93.00%(0.0019)
		$p = 0.02$															
		Individual												Combined			
		B-1				B-2				B-3							
$\delta_0 = \delta_1$	J	Est	Bias	Var	CP(ACIL)	Est	Bias	Var	CP(ACIL)	Est	Bias	Var	CP(ACIL)	Est	Bias	Var	CP(ACIL)
0.90	1	1.9604	-0.0396	0.1777	93.50%(0.0163)	1.9406	-0.0594	0.1746	94.00%(0.0162)	1.9612	-0.0388	0.2079	91.50%(0.0169)	1.9878	-0.0122	0.0784	95.50%(0.0116)
	2	2.0072	0.0072	0.0574	94.00%(0.0095)	2.0030	0.0030	0.0584	95.00%(0.0095)	2.0109	0.0109	0.0614	94.50%(0.0097)	2.0062	0.0062	0.0361	94.50%(0.0076)
	5	1.9973	-0.0027	0.0159	92.00%(0.0048)	1.9951	-0.0049	0.0163	93.50%(0.0048)	1.9955	-0.0045	0.0159	94.50%(0.0048)	1.9934	-0.0066	0.0126	93.50%(0.0041)
0.95	1	1.9935	-0.0065	0.0910	95.00%(0.0119)	1.9810	-0.0190	0.0847	95.00%(0.0120)	1.9928	-0.0072	0.0904	96.00%(0.0124)	2.0072	0.0072	0.0580	94.00%(0.0096)
	2	2.0133	0.0133	0.0316	94.50%(0.0070)	2.0076	0.0076	0.0312	94.00%(0.0070)	2.0090	0.0090	0.0317	94.50%(0.0071)	2.0069	0.0069	0.0240	95.50%(0.0061)
	5	1.9972	-0.0028	0.0105	93.50%(0.0038)	1.9960	-0.0040	0.0108	93.00%(0.0038)	1.9957	-0.0043	0.0107	93.00%(0.0038)	1.9977	-0.0023	0.0091	94.00%(0.0035)
1.00	1	1.9994	-0.0006	0.0242	94.00%(0.0061)	1.9994	-0.0006	0.0243	94.00%(0.0061)	1.9996	-0.0004	0.0243	94.00%(0.0061)	2.0127	0.0127	0.0229	94.50%(0.0060)
	2	2.0036	0.0036	0.0145	95.00%(0.0044)	2.0042	0.0042	0.0144	95.50%(0.0044)	2.0039	0.0039	0.0146	95.00%(0.0044)	2.0134	0.0134	0.0136	93.50%(0.0043)
	5	1.9997	-0.0003	0.0057	94.50%(0.0029)	1.9991	-0.0009	0.0058	94.00%(0.0029)	1.9984	-0.0016	0.0058	94.00%(0.0029)	2.0049	0.0049	0.0054	92.50%(0.0028)

TABLE 5 Simulation results for the AUC estimator based on the group and *random individual testing* approaches: estimate (Est), bias (Bias) and variance (Var), coverage probability (CP) and average confidence interval length (ACIL) of the estimators for the individual (B-1, B-2, B-3) and combined biomarkers. Entries of Var are multiplied 10 for better presentation. p is the prevalence, δ_0 and δ_1 are specificity and sensitivity, J is the group size, and B-1, B-2, B-3 stand for the individual biomarker 1, 2, 3, respectively.

		$p = 0.01$															
		Individual												Combined			
		B-1				B-2				B-3							
$\delta_0 = \delta_1$	J	Est	Bias	Var	CP(ACIL)	Est	Bias	Var	CP(ACIL)	Est	Bias	Var	CP(ACIL)	Est	Bias	Var	CP(ACIL)
0.90	1	0.8413	0.0175	0.0859	84.04%(0.3190)	0.8250	0.0277	0.0874	81.38%(0.3149)	0.7928	0.0250	0.1258	81.91%(0.3681)	0.8795	0.0216	0.0231	79.79%(0.1656)
	2	0.8263	0.0025	0.0446	90.95%(0.2433)	0.8109	0.0136	0.0491	86.43%(0.2470)	0.7830	0.0152	0.0576	88.44%(0.2883)	0.8687	0.0108	0.0151	89.45%(0.1400)
	5	0.8255	0.0017	0.0149	93.97%(0.1561)	0.7983	0.0010	0.0166	94.47%(0.1590)	0.7700	0.0022	0.0245	92.96%(0.1874)	0.8618	0.0039	0.0042	95.98%(0.0921)
0.95	1	0.8298	0.0060	0.0634	91.19%(0.2823)	0.8097	0.0124	0.0726	87.56%(0.2912)	0.7849	0.0171	0.0693	89.12%(0.3242)	0.8725	0.0146	0.0171	86.01%(0.1593)
	2	0.8304	0.0066	0.0235	91.50%(0.1994)	0.7986	0.0013	0.0250	93.00%(0.2053)	0.7694	0.0016	0.0312	93.00%(0.2220)	0.8655	0.0076	0.0110	89.50%(0.1249)
	5	0.8262	0.0024	0.0084	93.50%(0.1208)	0.8013	0.0040	0.0119	93.00%(0.1225)	0.7718	0.0040	0.0145	93.00%(0.1444)	0.8603	0.0024	0.0036	91.00%(0.0755)
1.00	1	0.8273	0.0035	0.0088	90.00%(0.1029)	0.8044	0.0071	0.0080	92.00%(0.1121)	0.7674	-0.0004	0.0083	94.00%(0.1141)	0.8633	0.0054	0.0032	92.50%(0.0737)
	2	0.8227	-0.0011	0.0049	96.00%(0.0874)	0.7992	0.0019	0.0057	96.00%(0.0947)	0.7684	0.0006	0.0069	93.50%(0.0991)	0.8595	0.0016	0.0031	90.00%(0.0614)
	5	0.8241	0.0003	0.0034	96.50%(0.0748)	0.7944	-0.0029	0.0041	95.50%(0.0803)	0.7676	-0.0002	0.0062	92.50%(0.0925)	0.8588	0.0009	0.0015	92.00%(0.0478)
		$p = 0.02$															
		Individual												Combined			
		B-1				B-2				B-3							
$\delta_0 = \delta_1$	J	Est	Bias	Var	CP(ACIL)	Est	Bias	Var	CP(ACIL)	Est	Bias	Var	CP(ACIL)	Est	Bias	Var	CP(ACIL)
0.90	1	0.8384	0.0146	0.0407	91.00%(0.2319)	0.8158	0.0185	0.0454	87.50%(0.2377)	0.7841	0.0163	0.0567	87.50%(0.2550)	0.8660	0.0081	0.0105	91.50%(0.1326)
	2	0.8262	0.0024	0.0153	93.00%(0.1534)	0.7992	0.0019	0.0160	94.00%(0.1566)	0.7710	0.0032	0.0229	90.00%(0.1767)	0.8606	0.0027	0.0061	91.50%(0.0940)
	5	0.8267	0.0029	0.0042	96.00%(0.0927)	0.8002	0.0029	0.0065	94.50%(0.0965)	0.7669	-0.0009	0.0066	95.50%(0.1112)	0.8595	0.0016	0.0016	95.00%(0.0537)
0.95	1	0.8283	0.0045	0.0229	92.00%(0.1821)	0.8022	0.0049	0.0224	94.50%(0.1906)	0.7710	0.0032	0.0234	93.50%(0.2064)	0.8620	0.0041	0.0082	91.50%(0.1150)
	2	0.8233	-0.0005	0.0076	96.50%(0.1187)	0.7989	0.0016	0.0116	90.00%(0.1209)	0.7721	0.0043	0.0110	93.50%(0.1304)	0.8603	0.0024	0.0042	93.50%(0.0770)
	5	0.8242	0.0004	0.0039	92.00%(0.0749)	0.7992	0.0019	0.0038	93.50%(0.0786)	0.7689	0.0011	0.0050	94.50%(0.0908)	0.8593	0.0014	0.0013	94.50%(0.0444)
1.00	1	0.8271	0.0033	0.0038	91.00%(0.0733)	0.8017	0.0044	0.0036	94.00%(0.0797)	0.7672	-0.0006	0.0046	94.00%(0.0816)	0.8598	0.0019	0.0020	94.50%(0.0526)
	2	0.8241	0.0003	0.0026	94.50%(0.0630)	0.7989	0.0016	0.0030	93.00%(0.0683)	0.7684	0.0006	0.0033	94.00%(0.0725)	0.8596	0.0017	0.0011	94.00%(0.0423)
	5	0.8247	0.0009	0.0021	91.00%(0.0530)	0.7985	0.0012	0.0025	94.00%(0.0574)	0.7670	-0.0008	0.0027	93.00%(0.0658)	0.8582	0.0003	0.0008	93.50%(0.0329)

TABLE 6 Simulation results for the AUC estimator based on the group approaches regarding log-normal (Log-normal) & gamma (Gamma) data as Normal data: estimate (Est), bias (Bias) and absolute value of relative error (|Error|) of the estimators for the combined biomarkers. N is the sample size, p is the prevalence, δ_0 and δ_1 are the specificity and sensitivity, J is the group size.

		Log-normal											
		N = 150000						N = 20000					
		p = 0.02			p = 0.05			p = 0.02			p = 0.05		
$\delta_0 = \delta_1$	J	Est	Bias	|Error|	Est	Bias	|Error|	Est	Bias	|Error|	Est	Bias	|Error|
0.90	1	0.8124	-0.0512	5.93%	0.7770	-0.0866	10.03%	0.8015	-0.0621	7.19%	0.7702	-0.0934	10.81%
	2	0.8126	-0.0510	5.99%	0.7775	-0.0861	9.96%	0.8045	-0.0591	6.84%	0.7746	-0.0890	10.30%
	5	0.8111	-0.0525	6.08%	0.7788	-0.0848	9.81%	0.8061	-0.0575	6.66%	0.7769	-0.0867	10.03%
0.95	1	0.8101	-0.0535	6.20%	0.7727	-0.0909	10.52%	0.8061	-0.0575	6.65%	0.7678	-0.0958	11.10%
	2	0.8117	-0.0519	6.00%	0.7752	-0.0884	10.23%	0.8064	-0.0572	6.62%	0.7699	-0.0937	10.85%
	5	0.8133	-0.0503	5.82%	0.7806	-0.0829	9.60%	0.8050	-0.0586	6.78%	0.7744	-0.0892	10.33%
1.00	1	0.7231	-0.1405	16.26%	0.7091	-0.1545	17.89%	0.7184	-0.1452	16.80%	0.7050	-0.1586	18.36%
	2	0.7997	-0.0639	7.39%	0.7398	-0.1238	14.34%	0.7927	-0.0709	8.21%	0.7371	-0.1265	14.65%
	5	0.8045	-0.0591	6.85%	0.7512	-0.1124	13.02%	0.7985	-0.0651	7.53%	0.7488	-0.1148	13.29%

		Gamma											
		N = 150000						N = 20000					
		p = 0.02			p = 0.05			p = 0.02			p = 0.05		
$\delta_0 = \delta_1$	J	Est	Bias	|Error|	Est	Bias	|Error|						
0.90	1	0.8640	0.0350	4.22%	0.8812	0.0522	6.30%	0.8645	0.0355	4.28%	0.8837	0.0547	6.60%
	2	0.8645	0.0355	4.28%	0.8805	0.0515	6.22%	0.8641	0.0351	4.24%	0.8798	0.0508	6.13%
	5	0.8760	0.0470	5.68%	0.8828	0.0538	6.49%	0.8777	0.0487	5.88%	0.8823	0.0533	6.43%
0.95	1	0.8725	0.0435	5.26%	0.8614	0.0324	3.91%	0.8775	0.0485	5.86%	0.8615	0.0325	3.92%
	2	0.8754	0.0464	5.60%	0.8697	0.0407	4.92%	0.8728	0.0438	5.29%	0.8710	0.0420	5.07%
	5	0.8736	0.0446	5.39%	0.8743	0.0453	5.47%	0.8738	0.0448	5.41%	0.8700	0.0410	4.95%
1.00	1	0.8064	-0.0226	2.73%	0.8063	-0.0227	2.74%	0.8063	-0.0227	2.73%	0.8060	-0.0230	2.77%
	2	0.8376	0.0086	1.04%	0.8337	0.0047	0.57%	0.8399	0.0109	1.32%	0.8331	0.0041	0.50%
	5	0.8597	0.0307	3.71%	0.8661	0.0371	4.48%	0.8579	0.0289	3.49%	0.8671	0.0381	4.60%
